# Demographic, Disease, and Treatment Characteristics of Primary Tracheal Cancers

**DOI:** 10.1245/s10434-024-16520-1

**Published:** 2024-11-18

**Authors:** Kristen Armel, Taylor Stamey, Joseph Maitre, Andrew R. Cunningham, Andrew W. Ju, Aidan Burke, James E. Speicher, Musharraf Navaid, Arjun Bhatt, Michael C. Larkins

**Affiliations:** 1 Brody School of Medicine (BSOM), East Carolina University (ECU), Greenville, NC USA; 2 Department of Radiation Oncology, BSOM, ECU, Greenville, NC USA; 3 Department of Cardiovascular Sciences, BSOM, ECU, Greenville, NC USA; 4 Division of Hematology/Oncology, Department of Internal Medicine, BSOM, ECU, Greenville, NC USA; 5 Department of Emergency Medicine, Boonshoft School of Medicine at Wright State University, Fairborn, OH USA

**Keywords:** Primary tracheal cancer, Squamous cell carcinoma, Adenoid cystic carcinoma, Cancer epidemiology, Chemotherapy, Radiotherapy, Surgical oncology

## Abstract

**Background:**

Primary tracheal cancers (PTCs) are rare neoplasms underreported in the literature. No consensus guidelines exist for the treatment of these cancers and multimodal management of these cancers has not been adequately explored for cases diagnosed over the past 2 decades.

**Methods:**

The Surveillance, Epidemiology, and End Results (SEER) database was queried to identify patients with PTC. Cox proportional hazards and log-rank testing was used to assess the association between demographic and treatment variables and 5-year cause-specific survival (CSS).

**Results:**

Among the 689 identified patients, age < 65 years at diagnosis (hazard ratio [HR] 0.64, *p* < 0.001), non-squamous cell carcinoma (SCC) histology (HR 0.22, *p* < 0.001), and treatment with surgery (HR 0.43, *p* < 0.001) were all associated with increased 5-year CSS. Regarding disease histology, patients with adenoid cystic carcinomas (ACCs) had increased 5-year CSS compared with those with neither SCC nor ACC histology and those with SCC histology (83.4% [76.0%, 90.8%] versus 50.3% [42.5%, 58.1%] and 28.8% [23.2%, 34.4%]; *p* < 0.001) based on univariate analysis. Despite the improved CSS associated with surgery, 55% of the identified cohort did not undergo surgery, with only 5.5% of these patients having ACC compared with 58% having SCC (*p* < 0.001).

**Conclusions:**

Age < 65 years, ACC histology, and treatment with surgery were associated with improved 5-year CSS among patients with PTC, although the significant proportion of this group not receiving surgery represents an opportunity for improved outcomes.

**Supplementary Information:**

The online version contains supplementary material available at 10.1245/s10434-024-16520-1.

Primary tracheal cancers (PTCs) are rare neoplasms, accounting for 0.2% of malignant neoplasms of the respiratory tract and with an incidence of only one per one million individuals.^1^ Primary tracheal tumors have been reported to present as locally advanced more often than as localized disease.^2,3^ The majority of these tumors are with squamous cell carcinomas (SCC) or adenoid cystic carcinomas (ACC), with ACC conferring a greater 5-year overall survival (OS) compared with SCC.^2^ Data regarding the treatment of PTC and the associated outcomes using evolving techniques of surgery, radiation, and chemotherapy are limited. Current studies suggest that surgical resection with primary reconstruction is the best treatment for this malignant cancer, although it has also been reported that fewer than half of patients with resectable tumors actually undergo surgical resection.^4^ The literature suggests surgical resection is the recommended treatment for most PTCs, although the role of other modalities, particularly radiotherapy (RT), have been explored to a very limited extent.^2–4^ Furthermore, multimodal management has not been investigated among patients in the United States (US). No consensus guidelines exist for the management of these cancers in general. The primary aim of this study was to evaluate demographic, disease, and treatment characteristics that may impact survival outcomes of patients with PTC in the US.

Given the rarity of these cancers and a lack of published analyses on the topic, knowledge concerning the demographic and treatment characteristics of PTC is limited. The most recent analysis of these cancers in the US (by Urdaneta et al.^2^) concerned patients from 1973 to 2004, and was limited in terms of the treatment characteristics analyzed. We sought to address this gap in the literature by providing a comprehensive epidemiological analysis of the population who have been diagnosed with PTC. Using the Surveillance, Epidemiology, and End Results (SEER) database, we also captured treatment data and measured survival endpoints, comparing different treatments received to evaluate for and to what extent benefit exists.

## Methods

### Data Source and Patient Selection

A retrospective cohort study utilizing the SEER program was conducted for patients with a diagnosis of PTC. Specifically, the 2000–2018 incidence dataset (April 2021 release) was queried via a case listing session in SEER*Stat.^5^ Inclusion criteria were primary site code C339 (corresponding to the trachea) and International Classification of Diseases for Oncology, 3rd edition (ICD-O-3) behavior code equal to ‘Malignant’. Patients with inadequate follow-up or cause of death (COD), as well as those under the age of 10 years at diagnosis, were further excluded from analysis.

### Variables/Outcome Data and Analysis

Demographics, summary staging, disease grade, treatment information, and survival from diagnosis were collected. Data were analyzed in SPSS version 29.0 (IBM Corporation, Armonk, NY, USA), with a *p*-value of <0.05 as the cut-off for statistical significance. Ninety-five percent confidence intervals (CIs) are reported in brackets, and Chi-square and Fisher’s exact tests are reported with exact two-sided *p*-values for categorical variables. Log-rank and Cox regression analyses were used to evaluate patient 5-year cause-specific survival (CSS). Forest plot creation was performed in Microsoft Excel version 2401 (Microsoft Corporation, Redmond, WA, USA).

## Results

### Population Attributes and Multivariate Analysis

Initially, 943 patients with a diagnosis of PTC were identified; after removing incomplete entries and patients with unknown survival data based on the flowchart in Fig. 1 of the electronic supplementary material (ESM), a final cohort of 689 patients was identified.

Five-year CSS for the entire cohort was 45.5% [41.3%, 49.7%], with a mean and median CSS of 2.9 and 3.3 years, respectively. The median patient age was 65–69 years. The distribution of patient age in 5-year brackets (as originally reported from the SEER database) can be found in Fig. 2 of the ESM. A summary of demographic, disease, and treatment characteristics can be found in Table 1.

**Table 1:** Demographic, treatment, and clinical characteristics of patients diagnosed with PTC between 2000 and 2020 identified via the SEER program

Variable	Total [*n* = 689] (%)
Age, years	
< 65	327 (47.5)
≥ 65	362 (52.5)
Sex	
Male	404 (58.6)
Female	285 (41.4)
Race	
Asian or Pacific Islander	44 (6.4)
Black	95 (13.8)
White	550 (79.8)
Marital status	
Married	371 (53.8)
Not married^a^	271 (39.3)
Unknown	47 (6.8)
Histology	
Adenoid cystic carcinoma	117 (17.0)
Squamous cell carcinoma	362 (52.5)
Other	210 (30.5)
Grade	
I (well-differentiated)	46 (6.7)
II (moderately differentiated)	151 (21.9)
III (poorly differentiated)	139 (20.2)
IV (undifferentiated, anaplastic)	26 (3.8)
Not applicable/incomplete entries	327 (47.5)
Stage	
Local	241 (35.0)
Regional	198 (28.7)
Distant	80 (11.6)
Incomplete entries	170 (24.7)
Lymph nodes surgically removed and pathologically examined	
Yes	91 (13.2)
No	494 (71.7)
Aspiration performed on regional lymph nodes	16 (2.3)
Unknown	88 (12.8)
Lymph nodes positive on pathologic examination	
Positive on examination	28 (4.1)
Negative on examination/no examination	559 (81.1)
Positive after aspiration	13 (1.9)
Unknown	89 (12.9)
Treatment	
Surgery only	114 (16.5)
CTX only	21 (3.0)
RT only	39 (5.7)
CTX and RT	121 (17.6)
Surgery with adjuvant CTX	8 (1.2)
Surgery with adjuvant RT	101 (14.7)
Surgery with neoadjuvant CTX	1 (0.1)
Surgery with neoadjuvant RT	5 (0.7)
Surgery with both adjuvant CTX and RT	54 (7.8)
Surgery with neoadjuvant RT and adjuvant CTX	1 (0.1)
Surgery with neoadjuvant and adjuvant RT	2 (0.3)
Surgery with neoadjuvant CTX and adjuvant RT	1 (0.1)
Surgery with neoadjuvant CTX and neoadjuvant RT	1 (0.1)
I ncomplete entries/no treatment	220 (31.9)
Surgery performed	
Yes	288 (41.8)
No/unknown	401 (58.2)
Chemotherapy administered	
Yes	208 (30.2)
No/unknown	481 (69.8)
Radiotherapy administered	
Yes	325 (47.2)
No/unknown	364 (52.8)

^a^Includes patients recorded as single, widowed, or divorced at the time of diagnosis

*PTC* primary tracheal cancer, *CTX* chemotherapy, *RT* radiotherapy

Results from multivariate analysis via Cox regression can be found in Table 2. In brief, age <65 years at diagnosis (hazard ratio [HR] 0.64, *p* < 0.001), non-SCC histology (HR 0.22, *p* < 0.001), and treatment with surgery (HR 0.43, *p* < 0.001) were all associated with increased 5-year CSS. Significant variance was seen between categories regarding patients with lymph nodes (LNs) surgically removed and pathologically examined (status = yes vs. no vs. unknown vs. aspirated LN-only; *p* = 0.049) and among the subset of patients with positive LNs after pathologic examination (status = yes vs. no vs. unknown vs. aspirated LN-positive; *p* < 0.001). Treatment with chemotherapy approached, but did not meet, statistical significance (HR 0.73, *p* = 0.052).

**Table 2:** Five-year cause-specific survival from multivariate Cox regression analysis of 689 patients identified with PTC.

Comparison	HR	*p*-Value
Age <65 years/age ≥65 years	0.64	<0.001
Sex	0.83	0.10
Race	NA	0.77
Marital status	NA	0.42
Histology	NA	<0.001
ACC and SCC/Other	0.34	<0.001
ACC and Other/SCC	0.22	<0.001
Grade	NA	0.15
Stage	NA	0.77
Lymph nodes pathologically examined^a^	NA	0.049
Lymph nodes positive^a^	NA	<0.001
Surgery (yes/no)	0.43	<0.001
Chemotherapy (yes/no)	0.73	0.05
Radiotherapy (yes/no)	0.82	0.21

For categorical variables with more than two categories, the overall comparison between all categories is listed in the columns to the right of the respective variable (in italics), while specific comparisons between groups within a categorical variable are listed below each variable. The groups corresponding to each variable can be found in Table 1

^a^Refers to lymph nodes surgically removed and sent for pathologic analysis

*HR* hazard ratio, *PTC* primary tracheal cancer, *ACC* adenoid cystic carcinoma, *SCC* squamous cell carcinoma, *NA* not available

### Univariate Analysis

Univariate analysis was subsequently conducted for those variables significant on multivariate analysis. Patients ≥65 years of age at diagnosis with PTC had a decreased 5-year CSS (34.2% [28.2%, 40.2%]) compared with those <65 years of age (56.9% [50.9%, 62.9%]; *p* < 0.001). Regarding disease histology, patients with ACC had increased 5-year CSS compared with those with neither SCC nor ACC histology, and those with SCC histology (83.4 % [76.0%, 90.8%] vs. 50.3% [42.5%, 58.1%] and 28.8% [23.2%, 34.4%]; *p* < 0.001).

Patients who had LNs examined had an associated increased 5-year CSS compared with those who did not (68.0 % [57.6%, 78.4%] vs. 42.2% [37.2%, 47.2%]) and among those with either known LN examination status (*n* = 88, 5-year CSS 43.2% [30.4%, 56.0%]) or who had LNs aspirated (*n* = 16, 5-year CSS 15.6% [0.0%, 41.6%]). Overall comparison among these groups was statistically significant (*p* < 0.001). Among those patients with LNs examined, obvious survival benefit was seen among those with negative or unknown pathology (5-year CSS 47.8% [43.0%, 52.6%] and 44.1% [31.5%, 56.7%], respectively) compared with those with positive pathology (18.1% [0.9%, 35.3%]) or positive LN aspiration (12.3% [0.0%, 34.5%]). Overall comparison among these groups was statistically significant (*p* = 0.019).

Finally, patients treated with surgery for PTC in general had associated increased 5-year CSS compared with those who did not (64.2% [57.8%, 70.6%] vs. 31.4% [26.0%, 36.8%]; *p* < 0.001). Analysis of 5-year CSS in the context of surgery and radiotherapy (RT) demonstrated survival benefit with treatment with either unimodal surgery or surgery in combination with RT (65.8% [56.4%, 75.2%] and 63.3% [54.9%, 71.7%], respectively) compared with unimodal RT (36.1% [27.7%, 44.5%]) and lack of treatment with surgery or RT (27.8% [21.0%, 34.6%]). Overall comparison among these groups was significant (*p* < 0.001).

Stratifying the analysis of surgery and RT by histology demonstrated no difference in 5-year CSS among cases of ACC (*p* = 0.10), although cases of SCC and those that were neither SCC nor ACC had significant variance among treatment modalities (*p* < 0.001 for both). Among cases of non-SCC and non-ACC PTC, surgical treatment was associated with obvious 5-year CSS benefit (5-year CSS 87.3% [76.5%, 98.1%]) compared with treatment with unimodal RT (41.4% [25.0%, 57.8%]; no difference in survival was seen between surgery and surgery in concert with RT (63.7% [43.1%, 84.3%]). Among cases of SCC, direct comparison between those treated with surgery and RT (40.4% [27.2%,53.6%]) and surgery alone (33.2% [17.4%,49.0%]) or RT alone (31.0% [21.0%,41.0%]) did not yield significant differences despite the overall trend being significant.

Information on the reason surgery was not performed in patients with PTC can be found in Table 2 of the ESM. Of those patients who did not receive surgery (55% of the entire cohort, *n* = 380), no difference was seen in CSS among the subgroups within this variable (*p* = 0.14). Despite comprising 17% of the entire patient cohort, patients diagnosed with ACC made up 5.5% of those not recommended for surgery (*n* = 17) compared with patients with SCC making up 58% (*n* = 180) and those with non-ACC and non-SCC histologies making up 34% (*n* = 106; *p* < 0.001 via Chi-square test). Additionally, male patients (59.1%) were more likely to not be recommended for surgery compared with female patients (40.9%; *p* < 0.001 via Fisher’s exact test). White patients (82.5%) were more likely to not be recommended for surgery than Black patients (12.7%) and patients identified as Asian or Pacific Islander (4.9%; *p* < 0.001 via Fisher’s exact test), although these relative percentages did not differ from the relative racial composition of the entire cohort (see Table 1).

## Discussion

### Variable Analysis

The only demographic factor that demonstrated an impact on 5-year OS was patient age, with those <65 years of age at diagnosis having increased survival compared with those ≥65 years of age (HR 0.64; *p* < 0.001). Similarly, univariate analysis demonstrated patients aged ≥65 years at diagnosis with PTC had a decreased 5-year CSS (34.2% [28.2%, 40.2%]) compared with those aged <65 years (56.9% [50.9%, 62.9%]; *p* < 0.001). These findings are similar to those of Hararah et al.,^6^ in which age <65 years at diagnosis was associated with increased 5-year all-cause mortality

Patients with ACC demonstrated increased survival compared with both SCC and all other PTC histologies (*p* < 0.001). On univariate analysis, patients with ACC demonstrated 5-year CSS of 83.4% compared with 50.3% and 28.8% for those with neither ACC nor SCC, and SCC histology, respectively. This difference is likely attributable to the aggressive nature of SCC, which is often found to be metastatic at the time of diagnosis.^7^Given the significant difference in survival and rate of disease progression, further analysis of tracheal carcinomas should differentiate ACC from SCC in the analysis, and further analysis among various histologies beyond ACC and SCC may be warranted.

Analysis of the distribution of patient ages concerning the type of PTC demonstrated a symmetric unimodal distribution pattern centered at 55 years for ACC compared with the left-skewed distribution favoring older age at diagnosis for SCC. This age distribution presents an intriguing insight into the clinical, pathogenic, and prognostic aspects of the disease. The left-skewed data observed for SCC suggests potential age-related risk factors contributing to its pathogenesis, such as smoking or environmental exposure.^8,9^ Conversely, the symmetric unimodal distribution of ACC across age groups implies other factors, perhaps unrelated to age or environmental exposures, may be at play. The possibility of genetic causes may be involved, although this is yet unproven, unlike with other early-onset cancers.^10,11^ The association between both the pathologic examination and subsequent positivity of surgically removed LNs is consistent with previous studies analyzing the effect of staging criteria on survival, which have found that positive LNs were correlated to worse survival.^12,13^

Currently, the treatment of choice for PTC is surgery for those whose tumors are found to be resectable.^14^ This process requires the involved portion of the trachea to be removed and reconstructed through primary anastomosis.^15^ In our analysis, patients treated surgically had increased 5-year OS on multivariate and univariate analysis. However, only 45% of the patients we identified underwent surgery, a finding similarly reported in previous literature.^14^ Further investigation into expanding the number of patients treated with surgery may be warranted, although it is unclear why patients were not recommended for surgery. Per the literature, those who are not surgical candidates currently have a 5-year survival rate of only 10% compared with over 50% for patients who underwent surgery.^16^ One line of thinking is that there may be barriers to surgical care, such as distance from academic centers where complicated procedures are carried out,^17^ although current surgical management of these may also be more conservative, secondary to the significant morbidity associated with surgical resection of the trachea, especially if the primary anastomosis fails.

RT has been shown to offer survival benefit among patients with both ACC and SCC of the trachea.^15,18^ These findings in the literature do not match our analysis, which demonstrated no significant improvement in 5-year CSS with treatment with RT (HR 0.82; *p* = 0.21). Furthermore, no difference in 5-year CSS was seen with adjuvant RT compared with unimodal surgery (65.8% vs. 63.3%), although both survival outcomes were improved compared with unimodal RT (5-year CSS 36.1%). Even stratifying by histology, obvious benefit was not seen with surgery and RT in combination compared with surgical treatment alone. Treatment with chemotherapy was not associated with improved OS in this analysis (HR 0.73; *p* = 0.05), although case reports have been published detailing the use of platinum-based therapies in the management of inoperable tracheal carcinomas.^19–22^ All of these findings based on treatment modality should be taken in the context of patient disease: patients with more advanced disease may indeed be treated more aggressively, however healthier patients may also be treated with more aggressive therapy such as RT before and after surgery, while older or more frail patients may not receive such treatment and outcomes may differ secondary to this.

### Limitations and Future Directions

While our study contributes substantial insights, it is not without limitations. The retrospective nature of our study relies on registry data, which may introduce biases. Our study is based on data from 2000 through 2018, over which time clinical guidelines, staging guidelines, and best practices have changed significantly. Changes in incidence, staging, and outcomes might be impacted due to these evolving stage definitions and practices. Several variables in the SEER database lack specificity such that it is not currently possible to evaluate specific differences in chemotherapy regimens. Furthermore, it is not known if specific treatments were carried out with palliative rather curative intent. Other variables missing from the SEER database include social history, history of human papilloma virus, and occupational exposures. Finally, outcomes related to morbidity, such as post-treatment scarring/fibrosis, etc., may be impacted by both demographic and treatment factors, as has been shown with head and neck cancer patients;^23^ however, these are not well-captured in the SEER database. Future research should explore these limitations and validate the findings presented in this analysis.

## Conclusions

We present a population-based analysis of primary tracheal carcinomas using the SEER program, which demonstrated patients aged <65 years at diagnosis, adenoid cystic histology, and surgical treatment improve 5-year OS on multivariate analysis. No obvious benefit was seen with surgical treatment in concert with RT compared with unimodal surgical treatment. A significant number of patients (55% of the entire cohort) did not undergo surgical treatment despite the improvement demonstrated in 5-year CSS, and these patients represent an opportunity for improved outcomes.

## Supplementary Information

Below is the link to the electronic supplementary material.

Supplementary file 1 (DOCX 119 KB)
