# Correction: Demographic, Disease, and Treatment Characteristics of Primary Tracheal Cancers

**DOI:** 10.1245/s10434-025-18192-x

**Published:** 2025-09-12

**Authors:** Kristen Armel, Taylor Stamey, Joseph Maitre, Andrew R. Cunningham, Andrew W. Ju, Aidan Burke, James E. Speicher, Musharraf Navaid, Arjun Bhatt, Michael C. Larkins

**Affiliations:** 1 Brody School of Medicine (BSOM), East Carolina University (ECU), Greenville, NC USA; 2 Department of Radiation Oncology, BSOM, ECU, Greenville, NC USA; 3 Department of Cardiovascular Sciences, BSOM, ECU, Greenville, NC USA; 4 Division of Hematology/Oncology, Department of Internal Medicine, BSOM, ECU, Greenville, NC USA; 5 Department of Emergency Medicine, Boonshoft School of Medicine at Wright State University, Fairborn, OH USA

**Correction to: Annals of Surgical Oncology (2024) 32:841-847** 10.1245/s10434-024-16520-1

The following disclaimer was missing from the original online version of this article. The original article was corrected:

The views and opinions expressed in this article/presentation are those of the author(s) and do not necessarily reflect official policy or position of the United States Air Force, Defense Health Agency, Department of Defense, or U.S. Government.

