# Supplementary material for: Demographic, Disease, and Treatment Characteristics of Primary Tracheal Cancers
Source: Ann Surg Oncol. 2024 Nov 18;32(2):841–7. doi: 10.1245/s10434-024-16520-1 (PMC11698749; doi:10.1245/s10434-024-16520-1)
Supplement: Supplementary file 1 — (DOCX 119 KB) [file 10434_2024_16520_MOESM1_ESM.docx]

**Supplemental Materials**

**Figure 1:** Distribution of Primary Tracheal Cancer Cases by Five-year Age Bracket


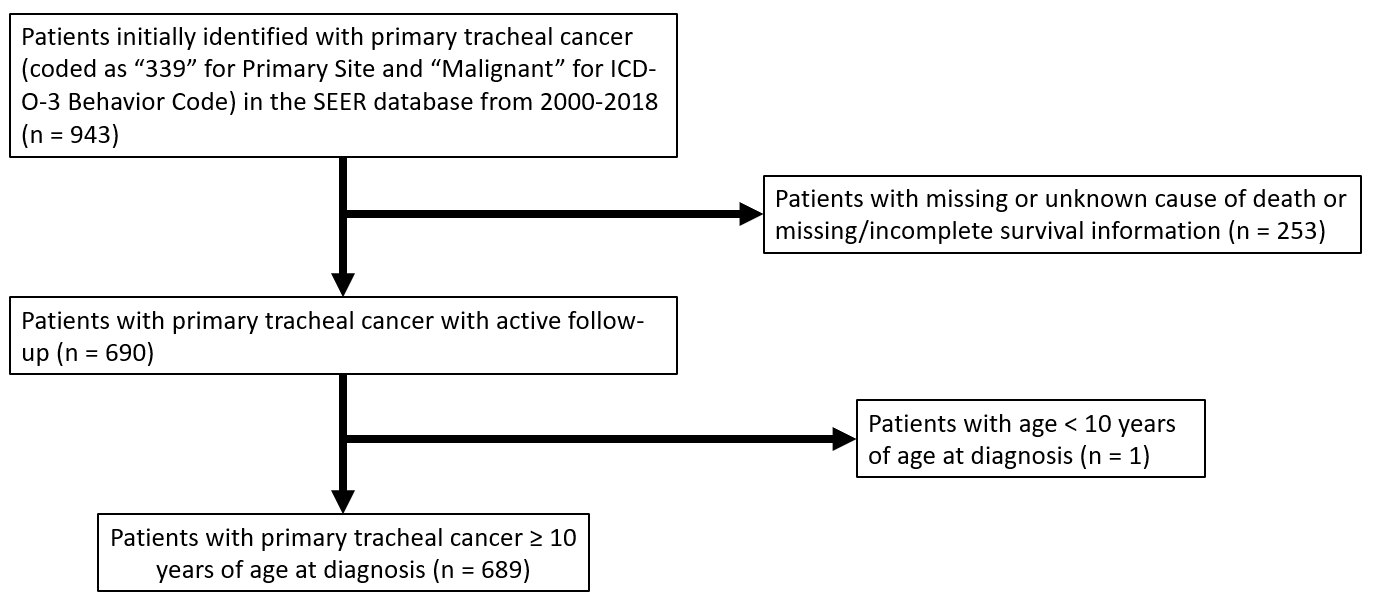


**Figure 1 Caption:** Flowchart depicting the selection of patients for analysis based on diagnosis with a primary tracheal cancer, as reported in the SEER database.

**Figure 2:** Distribution of Primary Tracheal Cancer Cases by Five-year Age Bracket


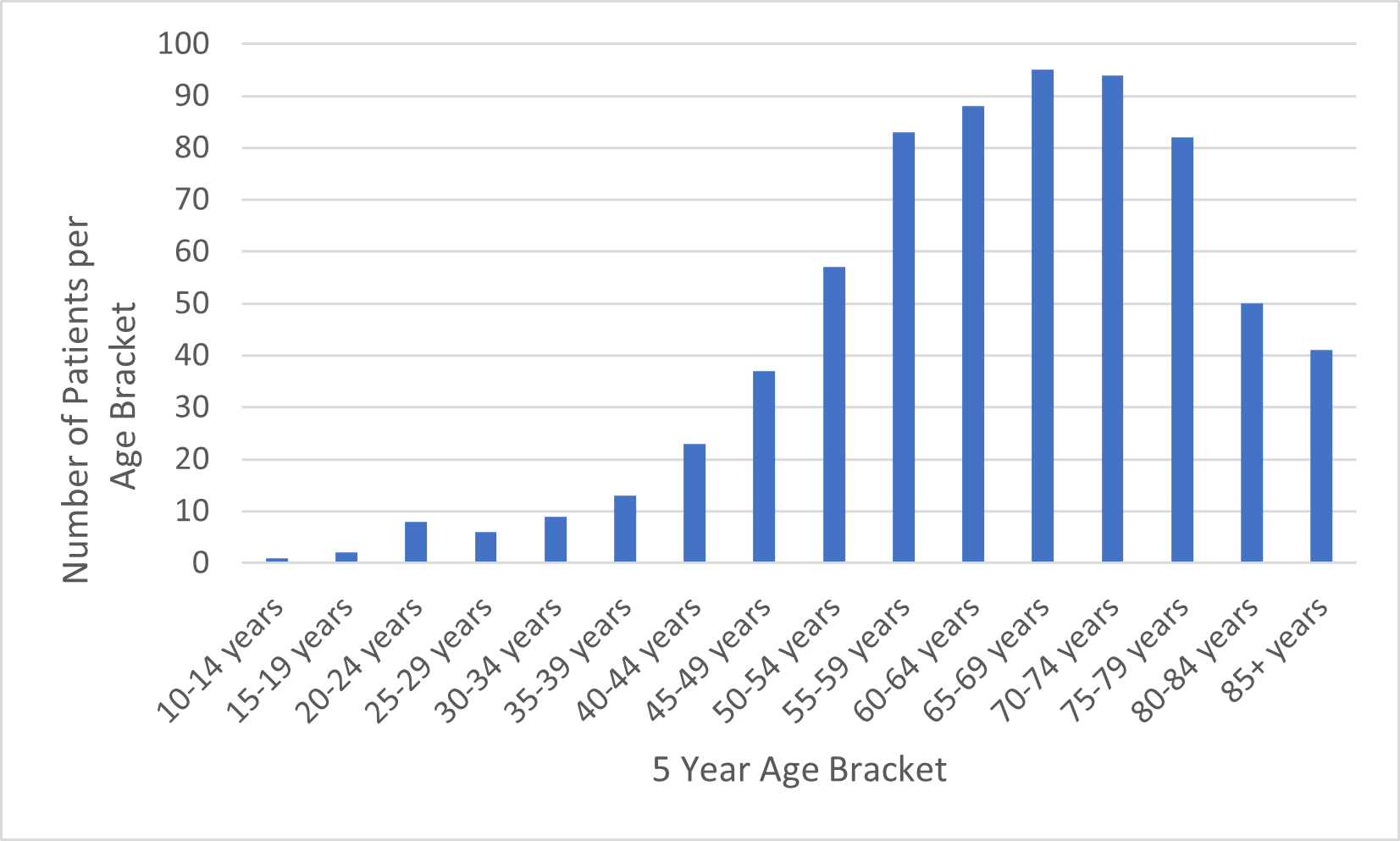


**Figure 2 Caption:** Distribution of primary tracheal cancers cases, as reported by the Surveillance, Epidemiology, and End Results (SEER) Program in five-year age brackets. A left skew can be appreciated, with more cases being diagnosed in older patients (median age was 65-69 years at diagnosis).

**Table 1:** Histologic Composition of Primary Tracheal Cancers

| **Histology (ICD-O-3 Code(s))** | **Total (n = 689, %) *** |
| --- | --- |
| Adenoid Cystic Carcinoma (8200) | 117 (17.0%) |
| Squamous Cell Carcinoma (8052, 8070-8072, 8074-8076, 8083) | 362 (52.5%) |
| Adenocarcinoma (8140, 8210, 8260, 8480) | 21 (3.0%) |
| Large Cell Carcinomas (8012, 8013, 8042) | 13 (1.9%) |
| Adenosquamous Carcinoma (8560) | 4 (0.6%) |
| Spindle Cell Carcinoma (8032) | 2 (0.3%) |
| Sarcomas (8033, 8800, 8803, 9040, 9043, 9120, 9220, 9231) | 22 (3.2%) |
| Small Cell Carcinomas (8041, 8044, 8045) | 31 (4.5%) |
| Non-Small Cell Carcinoma (8046) | 24 (3.5%) |
| Cribiform Carcinoma (8201) | 3 (0.4%) |
| Carcinoid Tumor (8240,8243,8249) | 9 (0.1%) |
| Neuroendocrine Tumors (8246) | 4 (0.6%) |
| Mucoepidermoid Tumor (8430) | 15 (2.2%) |
| Epithelial-myoepithelial Carcinoma (8562) | 2 (0.3%) |
| Thymic Carcinoma (8586) | 1 (0.1%) |
| Lymphomas (9590, 9673, 9680, 9699) | 26 (3.8%) |
| Malignant Fibrous Histiocytoma (8830) | 1 (0.1%) |
| Carcinoma in Pleomorphic Adenoma (8941) | 1 (0.1%) |
| Malignant Rhabdoid Tumor (8963) | 1 (0.1%) |
| Malignant Myoepithelioma (8982) | 1 (0.1%) |
| Teratoma, Malignant (9080) | 1 (0.1%) |
| Extraosseous Plasmacytoma (9734) | 1 (0.1%) |
| Other (8000, 8001, 8010) | 27 (3.9%) |

*Values rounded to nearest tenth of a percent

**Table 1 Caption:** Table depicting the histologic composition of all primary tracheal cancers identified via query of the Surveillance, Epidemiology, and End Results (SEER) Program. All cases were diagnosed between 2000 and 2018.

**Table 2:** Breakdown of Reason Why Surgery was not Performed

| **Variable** | **Total (n = 380, %)** |
| --- | --- |
| Not performed, patient died prior to surgery | 2 (0.5%) |
| Not recommended | 308 (81.1%) |
| Contraindicated due to other condition | 24 (6.3%) |
| Patient refused surgery | 9 (2.4%) |
| Unknown/Incomplete Entries | 37 (9.7%) |

**Table 2 Caption:** Reported reason why surgery was not performed among patients identified with primary tracheal cancer. Patients were identified via the SEER Program and were diagnosed between 2000 and 2018.
